# Establishment and Characterization of a High and Stable Porcine CD163-Expressing MARC-145 Cell Line

**DOI:** 10.1155/2018/4315861

**Published:** 2018-02-21

**Authors:** Xiangju Wu, Jing Qi, Xiaoyan Cong, Lei Chen, Yue Hu, Dongwan Yoo, Guisheng Wang, Fulin Tian, Feng Li, Wenbo Sun, Zhi Chen, Lihui Guo, Jiaqiang Wu, Jun Li, Jinbao Wang, Xiaomin Zhao, Yijun Du

**Affiliations:** ^1^Key Laboratory of Animal Biotechnology and Disease Control and Prevention of Shandong Province, College of Animal Science and Veterinary Medicine, Shandong Agricultural University, Tai'an 271018, China; ^2^Shandong Key Laboratory of Animal Disease Control and Breeding, Institute of Animal Science and Veterinary Medicine, Shandong Academy of Agricultural Sciences, Sangyuan Road No. 8, Jinan 250100, China; ^3^Department of Pathobiology, University of Illinois at Urbana-Champaign, 2001 South Lincoln Ave, Urbana, IL 61802, USA; ^4^Shandong Provincial Center for Animal Disease Control and Prevention, Jinan 250022, China; ^5^Department of Biology and Microbiology and Department of Veterinary and Biomedical Sciences, South Dakota State University, Brookings, SD 57007, USA

## Abstract

Isolation and identification of diverse porcine reproductive and respiratory syndrome viruses (PRRSVs) play a fundamental role in PRRSV research and disease management. However, PRRSV has a restricted cell tropism for infection. MARC-145 cells are routinely used for North American genotype PRRSV isolation and vaccine production. But MARC-145 cells have some limitations such as low virus yield. CD163 is a cellular receptor that mediates productive infection of PRRSV in various nonpermissive cell lines. In this study, we established a high and stable porcine CD163- (pCD163-) expressing MARC-145 cell line toward increasing its susceptibility to PRRSV infection. Indirect immunofluorescence assay (IFA) and Western blotting assays showed that pCD163 was expressed higher in pCD163-MARC cell line than MARC-145 cells. Furthermore, the ability of pCD163-MARC cell line to propagate PRRSV was significantly increased as compared with MARC-145 cells. Finally, we found that pCD163-MARC cell line had a higher isolation rate of clinical PRRSV samples and propagated live attenuated PRRS vaccine strains more efficiently than MARC-145 cells. This pCD163-MARC cell line will be a valuable tool for propagation and research of PRRSV.

## 1. Introduction

Porcine reproductive and respiratory syndrome (PRRS) first appeared in the late 1980s independently but almost simultaneously in North America and Europe. PRRS spread quickly to most swine producing countries worldwide and became one of the most economically important diseases in the swine industry [1]. The causative agent is PRRS virus (PRRSV) and two distinct genotypes are found: the European (EU) type (genotype 1) and the North American (NA) type (genotype 2) [2–4]. Sequence analysis shows they share approximately 60% nucleotide sequence identity at the genome level [5–8]. Since the emergence, they exhibit distinct genetic and antigenic variations and have been identified as dominating pathogens causing reproductive failures in sows and gilts, respiratory distress, high mortality rates for nursery pigs, and serious economic losses per year [9–12]. PRRSV was first isolated from fetuses suspicious of PRRS in 1995 in China [13]. In 2006, a large-scale devastating disease, known locally as “high fever,” broke out in China causing high morbidity of 50–100% and a mortality rate of 20–100% [14].

Pig is the only natural host of PRRSV. PRRSV has a restricted cell tropism for infection.* In vivo*, it replicates preferentially in porcine alveolar macrophages (PAMs) [15, 16].* In vitro*, PRRSV replication is limited to isolated PAMs [17], differentiated monocytes [18], and African green monkey kidney derived cells, such as MARC-145 [17, 18]. Cell tropism of PRRSV is determined by the interaction between the viral surface protein(s) and the cellular receptor(s) on the surface of host cells. Many cellular receptors have been described for PRRSV, including heparin sulfate (HS) [19], sialoadhesin (Sn) [20], vimentin [21], and CD151 [22]. However, there is no evidence that cells expressing these proteins* in trans* lead to a productive infection by PRRSV, nor any evidence is presented that endogenous expression of their cDNA confers susceptibility to nonpermissive cells. Since 2007, CD163, a cellular glycoprotein in the scavenger receptor cysteine-rich (SRCR) superfamily, has been described to function as a putative cellular receptor for PRRSV [23]. Furthermore, the expression of CD163 in nonpermissive cells such as PAM [24], CHO, and PK15 cells [25], BHK-21 cells [26], and murine macrophage-derived cells [27] has been shown to confer these cells to be permissive to PRRSV and support the production of PRRSV. The expression level of CD163 determines the level of PRRSV production, implicating that CD163 is a critical factor for PRRSV infection [20]. Other reports further indicate that the expression level of CD163 appears to correlate with the efficiency of PRRSV infection and an important residue of CD163 is found to be involved in the functional interaction with PRRSV [28, 29].

Isolation of PRRSV from clinical PRRSV samples is important for PRRSV diagnostics research. PAMs are susceptible to PRRSV but the primary cells are difficult to isolate from pigs and to maintain* in vitro*. MARC-145 cells are routinely used for NA genotype PRRSV isolation and vaccine production. However, MARC-145 cells have some limitations such as relatively low virus yield and low isolation rate of PRRSV from clinical specimen. In this study, a MARC-145 cell line stably and highly expressing pCD163 was generated and the susceptibility to PRRSV infection was evaluated. Remarkably, the expression of pCD163 in pCD163-MARC cells was robust, which suffices to render MARC-145 cells higher susceptible to clinical PRRSV isolation. The engineered cell line propagated live attenuated PRRSV vaccine strains more efficiently than MARC-145 cell line. As such, this pCD163-MARC cell line reported here will be a valuable tool to advance the biological characteristics analysis of PRRSV.

## 2. Materials and Methods

### 2.1. Animals

4- to 6-week-old PRRSV-negative pigs were obtained from a local farm without PRRSV, porcine circovirus 2, porcine parvovirus, pseudorabies virus, and* Actinobacillus pleuropneumoniae* history. All pigs were tested and proven to be seronegative for PRRS by indirect enzyme-linked immunosorbent assay (iELISA) and PRRSV negative by RT-qPCR.

### 2.2. Cells

PAMs were obtained from the lungs of PRRSV-negative pigs mentioned above. In brief, the lungs were washed five to eight times with sterilized phosphate-buffered saline (PBS) and each aliquot of washing fluid was centrifuged for 10 min at 1500 rpm. The resulting cell pellets were mixed together, washed again in PBS, and resuspended in RPMI 1640 medium (Invitrogen, Carlsbad, CA, USA) supplemented with 10% FBS, 2 mM L-glutamine, 1 mM nonessential amino acids, 100 U penicillin/ml, and 100 *μ*g streptomycin/ml. Cells were counted and seeded in 6-well tissue culture plates at a density of 2 × 10^6^ cells/well. MARC-145 cells were grown in Dulbecco's modified Eagle's medium (DMEM) supplemented with 10% FBS, 2 mM L-glutamine, 100 U of penicillin/ml, and 100 *μ*g of streptomycin/ml. These cells were cultured in a humidified incubator with 5% CO_2_ at 37°C.

### 2.3. Virus

NA genotype PRRSV classical strain S1 (GenBank accession number DQ459471) and highly pathogenic strain SY0608 (GenBank accession number EU144079) were kindly provided by Dr. Ping Jiang (Nanjing Agricultural University, China) [30, 31]. Both strains were propagated and titrated on MARC-145 cells and stored at −20°C for further use.

### 2.4. Construction of the pCI-pCD163 Plasmid

Based on pCD163 gene sequence (GenBank accession number HM991330), primers used for amplification of the full-length of pCD163 gene were designed. The total RNA was extracted from PAMs using TRIzol reagent according to the manufacturer's instructions (Invitrogen). RNA was further purified by using an RNeasy Minikit, including a DNase digestion step (Qiagen, Chatsworth, CA, USA). The purified RNA was reverse transcribed to cDNA using an oligo (dT) primer and SuperScript™ III Reverse Transcriptase (Invitrogen). The full-length of pCD163 gene was then amplified from cDNA using primer pair as follows: pCD163-Fwd (upstream primer): 5′-ATA*CTCGAG*CCACCATGGACAAACTCAGAATGGTGCTAC-3′ (containing* Xho*I site italicized); pCD163-Rev (downstream primer): 5′-ATA*GCGGCCGC*AAGCTTATCATTGTACTTCAGAG-3′ (containing* Not*I site italicized). Then the pCD163 gene was cloned into pCI-neo mammalian expression vector (Promega, Madison, WI, USA) using* Xho*I and* Not*I sites to obtain plasmid pCI-pCD163. The expression plasmid was sequenced to confirm the correct tandem in frame insertion of pCD163 gene.

### 2.5. Generation of pCD163-MARC Cell Line

MARC-145 cells were transfected with the plasmid pCI-pCD163 using the Lipofectamine 3000 reagent (Invitrogen) according to the manufacturer's protocol. pCD163-expressing cells were selected with 300 *μ*g/ml of G418 (Invitrogen) diluted in DMEM containing 10% FBS. The medium containing G418 was replenished every 3-4 days during selection. After selection, individual cell clone was isolated by a limited dilution method using 96-well cell culture plates, and fifteen resulting clones were screened for expression of pCD163 by Western blotting using mouse anti-pCD163 MAb (AbD Serotec, Raleigh, NC, USA). The cell clone expressing the highest level of pCD163 was chosen and designated as pCD163-MARC.

### 2.6. IFA

MARC-145 and pCD163-MARC cells were seeded directly onto coverslips. 48 h later, the expression of pCD163 was identified by IFA. IFA was also conducted to confirm the N protein expression of the 5th-passage PRRSV isolated from clinical specimen. Briefly, cells were washed twice in ice-cold phosphate-buffered saline (PBS) and fixed with 4% paraformaldehyde in PBS at 4°C for 1 h. Cells were then washed three times with ice-cold PBS and permeabilized with 0.5% Triton X-100 for 15 min. The coverslips were then incubated with mouse anti-pCD163 MAb (1 : 500) or anti-N MAb SDOW17 (Rural Technologies, Brookings, SD, USA) for 1 h. After three washes with PBS, coverslips were incubated with Alexa Fluor 488-conjugated goat anti-mouse IgG(H+L) antibody (Invitrogen) at room temperature for 1 h. Then the coverslips were washed three times and mounted with mounting buffer (60% glycerol and 0.1% sodium azide in PBS) and observed under an Olympus BX51 inverted fluorescence microscope.

### 2.7. Western Blotting Assay

After being grown in 6-well tissue culture plates for 48 h, MARC-145 and pCD163-MARC cells were lysed in ice-cold cell lysis buffer (Beyotime, Shanghai, China) containing 20 mM Tris (pH 7.5), 150 mM NaCl, and 1% Triton X-100 supplemented with phenylmethylsulfonyl fluoride (PMSF; Beyotime, Shanghai, China). The samples were analyzed by sodium dodecyl sulfate-polyacrylamide gel electrophoresis (SDS-PAGE) and Western blotting. Briefly, the samples were resolved in a 12% polyacrylamide gel. Separated proteins were then transferred onto a nitrocellulose membrane and probed with mouse anti-pCD163 MAb or *β*-actin antibody (Santa Cruz Biotechnology, Santa Cruz, CA, USA). Specific reaction products were detected with horseradish peroxidase- (HRP-) conjugated goat anti-mouse IgG (Boster, Wuhan, China). The membranes were developed using SuperSignal® West Pico Chemiluminescent Substrate according to the manufacturer's suggestions (Pierce, Rockford, IL, USA). Digital signal of chemiluminescent Western blotting was acquired by Bio-Rad GelDoc™ XR & ChemiDoc™ XRS system and analysis was conducted by the Quantity One program, version 4.6 (Bio-Rad). Each experiment was repeated three times.

### 2.8. Cell Proliferation Assay

Cell proliferation of the cell lines was evaluated as previously described [32] with the following modifications. Briefly, MARC-145 and pCD163-MARC cells were seeded in 24-well plates at a density of 1 × 10^4^ cells/well. Cells were digested and cell numbers were counted for a total of eight consecutive days to evaluate the proliferation rates. All assays were repeated at least three times, with each experiment performed in triplicate.

### 2.9. Growth Kinetics

The 60th passage of pCD163-MARC cells and MARC-145 cells was grown in 24-well plates to 80% confluency and infected with PRRSV classical strain S1 or highly pathogenic strain SY0608 at the same multiplicity of infection (MOI) of 1. Virus was allowed to adsorb for 1 h at 37°C. The inoculum was removed, and the cells were washed twice and then replaced with fresh medium. At 24 h, 48 h, 72 h, 96 h, and 120 h postinfection (hpi), the supernatants were collected. One part of culture supernatants was titrated by a microtitration infectivity assay on MARC-145 cells and the 50% tissue culture infective dose (TCID_50_) was calculated by the Reed–Muench method [33]. The remaining part of culture supernatants was subsequently used for SYBR Green real-time PCR assay. All assays were repeated at least three times, with each experiment performed in triplicate.

### 2.10. Real-Time RT-PCR

Real-time RT-PCR was carried out as described previously [34]. Total RNA of viral supernatants harvested as described above was extracted using TRIzol reagent. cDNA was synthesized using oligo d(T) primer as mentioned above. SYBR Green real-time PCR was performed to evaluate PRRSV RNA level, using the sequence of sense primer, 5′-AATAACAACGGCAAGCAGCAG-3′, and antisense primer, 5′-CCTCTGGACTGGTTTTGTTGG-3′. The cDNA was used as the template. The reaction was performed at 95°C for 2 min, followed by 40 cycles of 95°C for 15 s and 61°C for 1 min using the ABI 7300 detection system. For quantification, cDNA of PRRSV strain S1 was tenfold serially diluted and used to generate the standard curve. The real-time PCR method was very sensitive and could detect even 0.01 TCID_50_ of PRRSV in the viral supernatant. PRRSV RNA quantity of samples was determined by linear extrapolation of the Ct value plotted against the standard curve. All assays were repeated at least three times, with each experiment performed in triplicate.

### 2.11. PRRSV Isolation Assays

MARC-145 and pCD163-MARC cells were inoculated with each of 476 clinical tissue samples of lung, spleen, or lymph node which were PRRSV positively detected by RT-PCR (NA genotype) and kept in our laboratory from Jun 1, 2012, to Dec 31, 2016. The tissue samples were homogenized in PBS with 100 U of penicillin/ml and 100 *μ*g of streptomycin/ml and frozen and thawed three times and then clarified by centrifugation at 12,000*g* for 10 min at 4°C. The supernatants were collected, filtered by 0.22 *μ*m filter, and used to inoculate MARC-145 or pCD163-MARC cells. At 96 h postinoculation, the culture supernatants were collected and used to infect fresh MARC-145 cells or pCD163-MARC cells again. After passaging for another 3 times, the CPE of 5th-passage virus in MARC-145 and pCD163-MARC cells was observed and the PRRSV isolation rate was calculated.

### 2.12. Application to PRRS Vaccine Virus Production

MARC-145 and pCD163-MARC cells were cultured in roller bottles and infected with PRRSV R98 vaccine strain (GenBank accession number DQ355796, NA genotype classical strain) or PRRSV JXA1-R vaccine strain (GenBank accession number FJ548852, NA genotype highly pathogenic strain) at an MOI of 1. At 96 hpi, the virus titers in cell culture media were measured by a microtitration infectivity assay in MARC-145 cells. CPE in each MARC-145 cell culture well was observed microscopically for 5 days and the percentage of wells positive for CPE was calculated and recorded as TCID_50_/ml. All assays were repeated at least three times, with each experiment performed in triplicate.

### 2.13. Statistical Analysis

Data from MARC-145 and pCD163-MARC cells were compared and the differences were determined by one-way repeated measurement ANOVA and least significance difference (LSD). A* P* value < 0.05 was considered statistically significant [35].

## 3. Results

### 3.1. Generation and Characterization of the pCD163-MARC Cell Line

**Figure 1:**
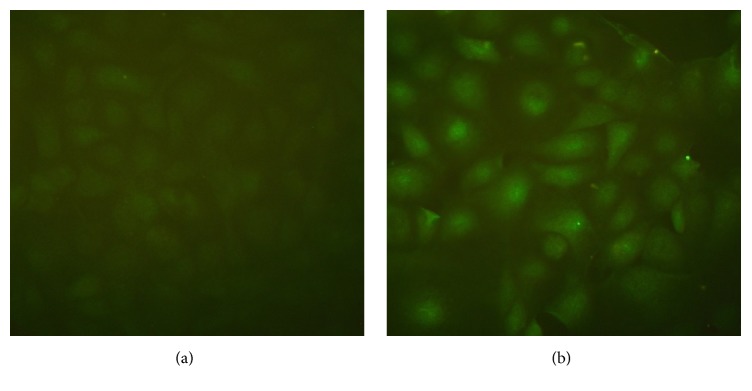
IFA analysis of the expression of pCD163 in MARC-145 (a) or pCD163-MARC cells (b) using mouse anti-pCD163 monoclonal antibody.

**Figure 2:**
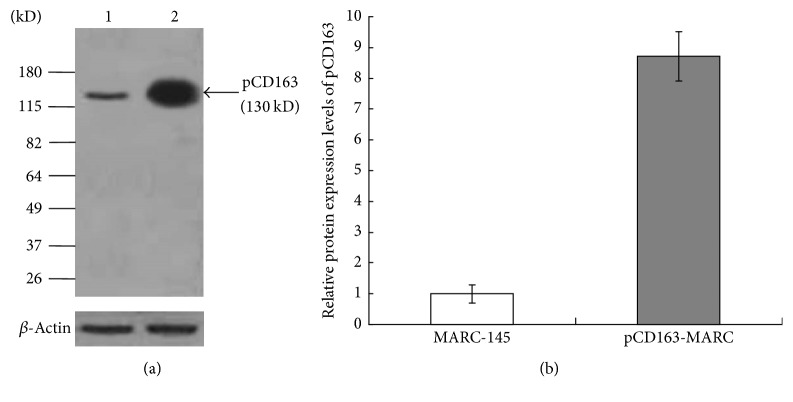
(a) Identification of the expression level of pCD163 in MARC-145 and pCD163-MARC cells by Western blotting. Anti-pCD163 antibody was used to detect the expression level of pCD163 in MARC-145 (lane 1) and pCD163-MARC cells (lane 2). *β*-Actin was used as loading control. The data presented here were results from one experiment of three Western blotting experiments. (b) Densitometry analysis of the digital image from three independent experiments. The band intensities were shown as the relative protein expression levels, normalized with *β*-actin. Error bars indicate the standard deviations of three experiments.

**Figure 3:**
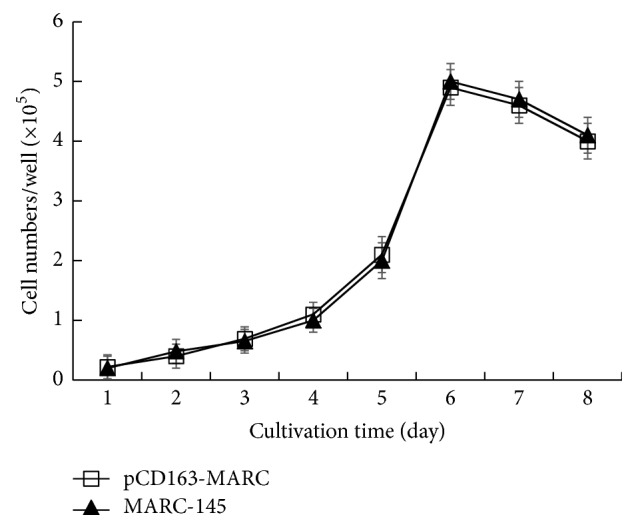
The growth curves of MARC-145 and pCD163-MARC cells. Cells were seeded at a concentration of 1 × 10^4^ cells/well and the average cell count at each time point was plotted against time. The data represent the means of three independent experiments, with each experiment performed in triplicate. Error bars indicate the standard deviations of three experiments.

The eukaryotic expression plasmid pCI-pCD163 was constructed and the sequencing data confirmed a 100% identity with pCD163 sequence (GenBank accession number HM991330), demonstrating a successful and correct insertion of pCD163 gene in the construct. The plasmid was then transfected into MARC-145 cells. After selection with G418, each cell clone was examined for pCD163 gene expression. The cell clone of the highest pCD163 expression was identified by IFA and Western blotting and designated pCD163-MARC. The fluorescence intensity of pCD163-MARC cells was significantly higher than that of MARC-145 cells, and pCD163 was present both on the cell surface and in cytoplasm (Figure 1). The pCD163 expression level was further examined by Western blotting (Figure 2(a)). pCD163-MARC cells were found to express 8.7 times higher level of pCD163 than MARC-145 cells (Figure 2(b)). The growth speed and viability of pCD163-MARC cells were compared with MARC-145 cells. As shown in Figure 3, pCD163-MARC cells had similar cell viability and doubling times with the parental MARC-145 cells (*P* > 0.05).

### 3.2. pCD163-MARC Cells Are Highly Susceptible to Both PRRSV Classical and Highly Pathogenic Strains

**Figure 4:**
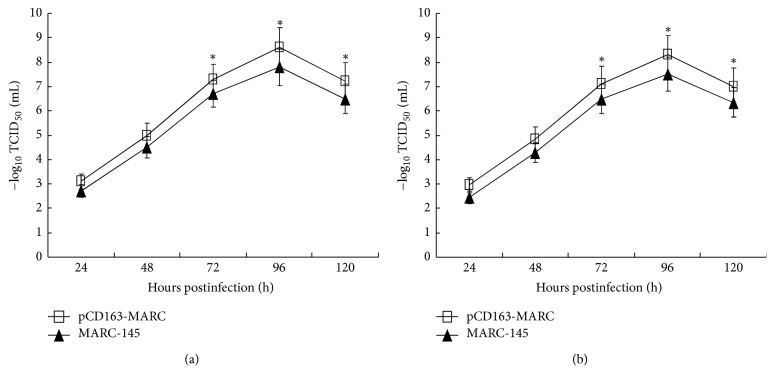
Growth kinetics of PRRSV in pCD163-MARC and MARC-145 cells. Growth curves of PRRSV classical strain S1 (a) and highly pathogenic strain SY0608 (b) were determined independently in pCD163-MARC and MARC-145 cells. pCD163-MARC and MARC-145 cells were individually infected with PRRSV at an MOI of 1 for 1 h. Culture supernatants were collected at the indicated times and virus titers were determined. The data represent the means of three independent experiments, with each experiment performed in triplicate. Error bars indicate the standard deviations of three experiments. ^*∗*^*P* < 0.05.

**Figure 5:**
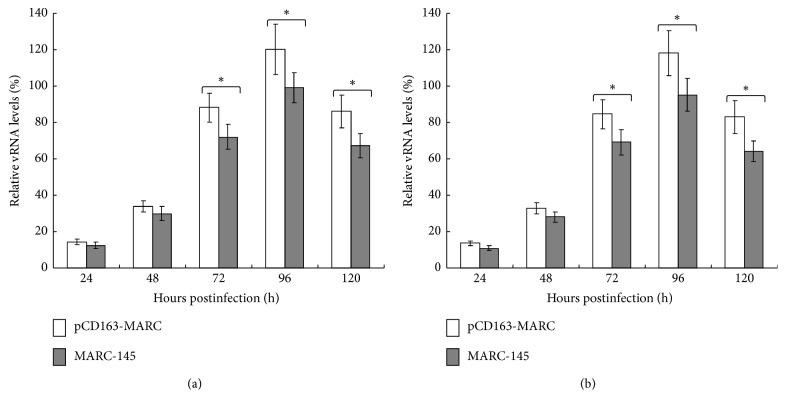
Real-time PCR analysis of PRRSV in pCD163-MARC and MARC-145 cells. pCD163-MARC and MARC-145 cells were individually infected with PRRSV classical strain S1 (a) and highly pathogenic strain SY0608 (b) at an MOI of 1 for 1 h. RNA was extracted from culture supernatants collected at the indicated times and subjected to SYBR Green real-time PCR. The relative PRRSV RNA levels of samples were determined by linear extrapolation of the Ct value plotted against the standard curve. The data represent the means of three independent experiments, with each experiment performed in triplicate. Error bars indicate the standard deviations of three experiments. ^*∗*^*P* < 0.05.

To evaluate the susceptibility of pCD163-MARC cells to PRRSV, 60th passage of pCD163-MARC and MARC-145 cells was individually infected with PRRSV classical strain S1 or highly pathogenic strain SY0608 at an MOI of 1. TCID_50_ was measured to determine viral growth kinetics. As shown in Figure 4, pCD163-MARC cells propagated both PRRSV S1 and SY0608 more efficiently than MARC-145 cells (*P* < 0.05). Real-time RT-PCR was also conducted to amplify the PRRSV N gene from the culture media at different time points. As shown in Figure 5, MARC-145 cells clearly contained lower levels of viral RNA than the pCD163-MARC cells (*P* < 0.05). In sum, the data suggested that pCD163-MARC cells are more susceptible to infections by both PRRSV S1 and SY0608 strains, thereby emphasizing that the expression level of pCD163 correlates well with the overall level of PRRSV propagation.

### 3.3. pCD163-MARC Cells Are More Suitable for Clinical PRRSV Isolation

**Figure 6:**
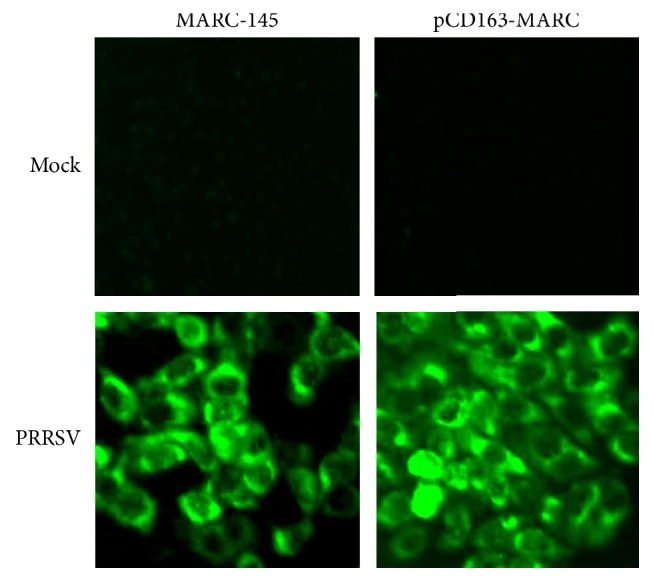
IFA analysis of N protein expression of 5th-passage PRRSV in MARC-145 and pCD163-MARC cells using anti-N MAb SDOW17.

Isolation and characterization of PRRSV from clinical specimen are an important part of PRRSV research and diagnostics. pCD163-MARC cells and MARC-145 cells were simultaneously compared to isolate PRRSV from 476 clinical PRRSV positive tissue samples collected from Jun 1, 2012, to Dec 31, 2016. As shown in Table 1, PRRSV isolation rate in the engineered pCD163-MARC cells was higher than that in MARC-145 cells, indicating that pCD163-MARC cell line was more suitable for clinical PRRSV isolation. The results of IFA further confirmed the N protein expression of 5th-passage virus in MARC-145 and pCD163-MARC cells, which was consistent with the observation of CPE (Figure 6).

### 3.4. pCD163-MARC Cells Are More Suitable for PRRSV Vaccine Production

Vaccination is considered the major protective measure to control PRRS. Next, we examined and compared pCD163-MARC and MARC-145 cells for their ability to propagate live attenuated PRRS vaccine strains. As summarized in Table 2, viral titers produced by pCD163-MARC cells for PRRSV R98 and JXA1-R vaccine strain were higher than MARC-145 cells (*P* < 0.05). This result supported a notion that pCD163-MARC cells are more effective in propagation of PRRSV vaccine strain and can be used in vaccine production.

## 4. Discussion

To date, the propagation of PRRSV using cell lines* in vitro* can be carried out in three methods. One method represents the monocyte-macrophage lineage containing PAMs and peripheral blood monocytes. Another method represents African green monkey kidney cell MA-104 and its derivatives MARC-145 and CL-2621. The third is recombinant cell lines created from PRRSV nonpermissive cells by recombinant DNA technology, which confers permissiveness to PRRSV infection after expressing the corresponding receptor [36]. So far, HS, Sn, CD163, CD151, and vimentin have been reported as PRRSV receptors. With the growth of CD163, the infection of PRRSV in cultured monocytes is regularly increasing [37]. The results of flow cytometry show a direct correlation between the expression of CD163 in PAM cells and PRRSV infectivity [38]. What is more, CD163 has been proved to be related to the entry of PRRSV into MARC-145 cells which do not belong to the monocyte-macrophage lineage [23]. Pretreatment of PAMs with tetradecanoyl phorbol acetate (TPA) or lipopolysaccharide (LPS) shows that the CD163 expression decreases, and accordingly infection of PRRSV is reduced [39]. Transfection of PRRSV nonpermissive cells with the CD163 gene derived from swine, canine, murine, human, and simian can render these cells permissive to PRRSV infection, fully confirming the function of CD163 [23, 39]. All of these data demonstrate that CD163 plays an important role in determining PRRSV susceptibility and productive infection. So far, numerous cell lines were constructed to enhance PRRSV replication, such as continuous PAM [24], CHO and PK15 cells [25], BHK-21 cells [26], and murine macrophage-derived cells [27], fully revealing the importance of CD163. MARC-145 cells are routinely used for NA genotype PRRSV isolation and vaccine production. However, MARC-145 cells have some limitations such as relatively low virus yield and no cell lines constructed based on MARC-145 cells to enhance the propagation of PRRSV. The current study has attempted to generate a subline of MARC-145 cells stably expressing pCD163, such that these cells can be used to facilitate sensitive isolation of PRRSV for research and diagnostics and to produce vaccine virus effectively.

CD163 is a 130 kD glycoprotein containing a large extracellular region of 9 SRCR domains, a single transmembrane (TM) domain, and a short cytoplasmic tail [40, 41]. Domains in pCD163 related to PRRSV infection have been identified using methods of various deletion and chimeric constructs. Touching the importance of SRCR domains, excluded are the first four N-terminal repeats (SRCRs 1–4), whereas SRCR5 is vital for mediating PRRSV infection [23, 39, 42]. Further studies demonstrated that the TM domain is required for PRRSV susceptibility. The cytoplasmic tail is conserved among species but is not required for PRRSV infection [43]. In our study, the full-length of pCD163 gene was amplified by RT-PCR from PAMs and cloned into the eukaryotic expression vector pCI-neo containing the geneticin gene for antibiotics selection. The plasmid pCI-pCD163 was introduced to MARC-145 cells and positive cells were selected based on the resistance to G418, and the highest level of pCD163 expressing cell clone was selected, and pCD163-MARC was established.

PRRSV was first isolated from fetuses suspicious of PRRS in 1995 [13], which belongs to NA genotype PRRSV [44]. Since then, PRRSV spread in China has been predominantly NA genotype. In 2006, highly pathogenic PRRS broke out in China and caused enormous economic losses [14]. In the present study, we used NA genotype PRRSV classical strain S1 and highly pathogenic strain SY0608 to represent PRRSV circulating in China and examined their propagation in the established pCD163-MARC cells. The growth kinetics of PRRSV and the determination of PRRSV RNA demonstrated that pCD163-MARC cells had higher production level of both PRRSV strains (Figures 4 and 5). The presenting data here show that pCD163 has a pivotal role in MARC-145 for PRRSV infection in accordance with previous reports that the expression level of pCD163 could determine the efficiency of PRRSV infection [23]. However, whether the cell line is used for EU genotype PRRSV isolation needs to be studied in the future.

Isolation of PRRSV from clinical samples is important for PRRSV research and diagnostics. The PRRSV isolation rate in pCD163-MARC cells reaches 98.2–100%, which is significantly higher than the isolation rate in MARC-145 cells (Table 1). We show that pCD163-MARC cells are more suitable for isolation of field viruses. Using these cells, more PRRSV isolates may be obtained to study their biological characteristics. In addition, two vaccine strains currently used in China can be produced at higher titers in pCD163-MARC cells than in MARC-145 cells, demonstrating the great potential of this cell line for efficient production of commercial vaccines (Table 2).

## 5. Conclusions

We have successfully established a high and stable pCD163 expressing MARC-145 cell line with increased susceptibility to PRRSV infection. This pCD163-MARC cell line will be a valuable tool not only to facilitate PRRSV propagation for PRRSV isolation and vaccine production, but also to study the biological characteristics of PRRSV.

## Figures and Tables

**Table 1:** PRRSV isolation rate of MARC-145 cells and pCD163-MARC cells.

Clinical PRRSV positive samples	PRRSV isolation rate	
	MARC-145	pCD163-MARC
Jun 1, 2012–Dec 31, 2012, 59 samples	88.1% (52/59)	100% (59/59)
Jan 1, 2013–Dec 31, 2013, 96 samples	86.5% (83/96)	100% (96/96)
Jan 1, 2014–Dec 31, 2014, 103 samples	86.4% (89/103)	99.0% (102/103)
Jan 1, 2015–Dec 31, 2015, 106 samples	85.8% (91/106)	99.1% (105/106)
Jan 1, 2016–Dec 31, 2016, 112 samples	85.7% (96/112)	98.2% (110/112)
Total PRRSV isolation rate	86.3% (411/476)	99.2% (472/476)

**Table 2:** Ability of pCD163-MARC and MARC-145 cells to produce PRRS vaccine virus.

Cells	Infected PRRSV strain	Harvest time	Virus titer^a^
pCD163-MARC	R98	96 h	1 × 10^8.8±0.2^ TCID_50_/mL^*∗*^
MARC-145	R98	96 h	1 × 10^8.0±0.3 ^ TCID_50_/mL
pCD163-MARC	JXA1-R	96 h	1 × 10^8.5±0.1^ TCID_50_/mL^*∗*^
MARC-145	JXA1-R	96 h	1 × 10^7.6±0.2^ TCID_50_/mL
